# Commentary on ‘Comparison of craniotomy and decompressive craniectomy for acute subdural hematoma - a meta-analysis of comparative study’

**DOI:** 10.1097/JS9.0000000000001711

**Published:** 2024-05-29

**Authors:** Ayush Anand, Kelechi M. Azode, Ghomsi M. Christelle Nathalie, Mahalaqua N. Khatib, Quazi S. Zahiruddin, Sarvesh Rustagi, Prakasini Satapathy

**Affiliations:** a B.P. Koirala Institute of Health Sciences, Dharan, Nepal; b Medical Laboratories Techniques Department, Al-Mustaqbal University, Hillah, Babil, Iraq; c Obafemi Awolowo University Teaching Hospitals Complex, Ife, Nigeria; d Department of Neurosurgery, Centre hospitalier universitaire de Yopougon, Abidjan, Cote d’Ivoire; e Division of Evidence Synthesis; f South Asia Infant Feeding Research Network (SAIFRN), Division of Evidence Synthesis, Global Consortium of Public Health and Research, Datta Meghe Institute of Higher Education, Wardha; g School of Applied and Life Sciences, Uttaranchal University, Dehradun, Uttarakhand; h Center for Global Health Research, Saveetha Medical College and Hospital, Saveetha Institute of Medical and Technical Sciences, Saveetha University, Chennai; i MediSurg Research, Darbhanga, India

*Dear Editor,*

The management of acute subdural hematoma (ASDH) remains a critical and contentious issue in neurosurgical practice^1^. The recent meta-analysis by Li *et al*.^2^ provides a comparative evaluation of craniotomy (CO) and decompressive craniectomy (DC) for the evacuation of ASDH, shedding light on the outcomes associated with these two surgical interventions.

The meta-analysis, encompassing 15 studies with a total of 4853 patients, presents several pivotal findings. First, the study reported that DC is associated with higher mortality (40.6%) compared to CO (31.5%). This aligns with previous studies suggesting a potential survival disadvantage for DC. Second, poorer neurological outcomes were more frequently observed in the DC group (72.7%) compared to the CO group (54.3%). This difference persisted across multiple functional outcome scales, reinforcing concerns about the long-term efficacy of DC in ASDH management. Third, when adjusting for baseline severity, the differences in mortality and neurological outcomes between the two procedures diminished. This suggests that preoperative patient characteristics significantly influence surgical outcomes, potentially overshadowing the direct effects of the surgical technique itself. Fourth, the analysis found no significant difference in reoperation rates between CO and DC, indicating that both procedures have comparable rates of surgical complications requiring additional interventions.

While the meta-analysis provides valuable insights, several limitations must be considered. First, the included studies exhibit significant heterogeneity in terms of design, patient populations, and clinical settings. This variability introduces challenges in pooling data and interpreting results. Second, the majority of the included studies are retrospective cohort studies, which are inherently prone to selection bias and confounding factors. This limits the robustness of the conclusions drawn. Third, only one randomized controlled trial (RCT) is included, which constrains the overall quality of the evidence. Fifth, the primary focus on mortality and neurological outcomes, while critical, does not encompass other relevant outcomes such as quality of life and long-term functional independence. Sixth, despite efforts to adjust for baseline severity, residual confounding cannot be entirely excluded. The complex interplay of multiple factors influencing surgical outcomes necessitates a cautious interpretation of the findings. Seventh, cost cost-effectiveness of both the interventions, along with the role of post-operative monitoring, is crucial^3,4^.

The findings of this meta-analysis have several implications for neurosurgical practice. First, the importance of baseline severity underscores the need for personalized treatment decisions. Surgeons should consider patient-specific factors, including preoperative Glasgow Coma Scale scores and the presence of brain swelling when choosing between CO and DC. Second, the observed mortality and functional outcome differences challenge the perceived superiority of DC in certain clinical scenarios. Surgeons should critically appraise the indications for each procedure and avoid a one-size-fits-all approach. Third, there is a clear need for more high-quality RCTs to validate these findings and inform evidence-based clinical guidelines. Collaborative multicenter trials could address the current evidence gaps and enhance the generalizability of results. Fourth, future research should incorporate a broader range of outcome measures, including quality of life and long-term functional status. This will provide a more comprehensive understanding of the impact of surgical interventions on patient recovery and well-being.

In conclusion, the meta-analysis by Li *et al*. contributes significantly to the ongoing debate on the optimal surgical management of ASDH. While it highlights important trends and associations, the limitations inherent in the current body of evidence call for cautious interpretation and application in clinical practice. Personalized patient care, critical appraisal of surgical techniques, and a commitment to high-quality research are essential to improving outcomes for patients with ASDH.

## Ethical approval

Ethical approval is not applicable for this correspondence article.

## Consent

Informed consent is not applicable for this correspondence article.

## Source of funding

None.

## Author contribution

A.A. and K.M.A.: conceptualization, project administration, supervision, validation, writing – original draft, and writing – review and editing; G.M.C.N.: supervision, validation, writing – original draft, and writing – review and editing; M.N.K., Q.S.Z., S.R., and P.S.: writing – original draft and writing – review and editing.

## Conflicts of interest disclosure

There are no conflicts of interest.

## Research registration unique identifying number (UIN)

Not applicable.

## Guarantor

Kelechi Michael Azode and Ghomsi M. Christelle Nathalie.

## Data availability statement

None.

## Provenance and peer review

Not commissioned, externally peer-reviewed.

## Assistance with the study

None.

## Presentation

None.

## References

[1] Ho T Frisbie J Wasfie T . A retrospective analysis of factors influencing readmission rates of acute traumatic subdural hematoma in the elderly: a cohort study. Int J Surg Open 2019;20:20–23.

[2] Li H Yao Y Gan W . Comparison of craniotomy and decompressive craniectomy for acute subdural hematoma - a meta-analysis of comparative study. Int J Surg 2024. 10.1097/js9.0000000000001590 PMC11326010 38884600

[3] Anand A Satapathy P Sharma RK . Redefining postoperative monitoring in chronic subdural hematoma: the promise of trans-burr hole sonography. Int J Surg Open 2024. [Epub ahead of print]. 10.1097/io9.0000000000000084

[4] Tong X Xue X Liu A . Comparative study on clinical outcomes and cost-effectiveness of chronic subdural hematomas treated by middle meningeal artery embolization and conventional treatment: a national cross-sectional study. Int J Surg 2023;109:3836–3847. 37830938 10.1097/JS9.0000000000000699 PMC10720801

